# Neuropsychological and Brain Volume Differences in Patients with Left- and Right-Beginning Corticobasal Syndrome

**DOI:** 10.1371/journal.pone.0110326

**Published:** 2014-10-30

**Authors:** Kerstin Jütten, Peter Pieperhoff, Martin Südmeyer, Axel Schleicher, Stefano Ferrea, Svenja Caspers, Karl Zilles, Alfons Schnitzler, Katrin Amunts, Silke Lux

**Affiliations:** 1 Institute of Neuroscience and Medicine (INM-1), Research Centre Jülich, Jülich, Germany; 2 Department of Neurology, Center for Movement Disorders and Neuromodulation, Medical Faculty, Heinrich-Heine-University, Düsseldorf, Germany; 3 Institute of Clinical Neuroscience and Medical Psychology, Medical Faculty, Heinrich-Heine-University, Düsseldorf, Germany; 4 JARA-Brain, Jülich-Aachen Research Alliance, Jülich, Germany; 5 Department of Psychiatry, Psychotherapy and Psychosomatics, RWTH Aachen University, Aachen, Germany; 6 C. & O. Vogt Institute for Brain Research, Heinrich Heine University, Düsseldorf, Germany; University of Ulm, Germany

## Abstract

**Background:**

Corticobasal Syndrome (CBS) is a rare neurodegenerative syndrome characterized by unilaterally beginning frontoparietal and basal ganglia atrophy. The study aimed to prove the hypothesis that there are differences in hemispheric susceptibility to disease-related changes.

**Methods:**

Two groups of CBS patients with symptoms starting either on the left or right body side were investigated. Groups consisted of four patients each and were matched for sex, age and disease duration. Patient groups and a group of eight healthy age-matched controls were analyzed using deformation field morphometry and neuropsychological testing. To further characterize individual disease progression regarding brain atrophy and neuropsychological performance, two female, disease duration-matched patients differing in initially impaired body side were followed over six months.

**Results:**

A distinct pattern of neural atrophy and neuropsychological performance was revealed for both CBS: Patients with initial right-sided impairment (r-CBS) revealed atrophy predominantly in frontoparietal areas and showed, except from apraxia, no other cognitive deficits. In contrast, patients with impairment of the left body side (l-CBS) revealed more widespread atrophy, extending from frontoparietal to orbitofrontal and temporal regions; and apraxia, perceptional and memory deficits could be found. A similar pattern of morphological and neuropsychological differences was found for the individual disease progression in l-CBS and r-CBS single cases.

**Conclusions:**

For similar durations of disease, volumetric grey matter loss related to CBS pathology appeared earlier and progressed faster in l-CBS than in r-CBS. Cognitive impairment in r-CBS was characterized by apraxia, and additional memory and perceptional deficits for l-CBS.

## Introduction

Corticobasal Syndrome (CBS) is a rare (prevalence 6: 100 000), rapidly progressing neurodegenerative syndrome characterized by brain atrophy in combination with motor and cognitive impairment [1], [2]. The disease starts around 60 years of age [3] and shows high inter-individual diversification regarding grey matter atrophy and behavioral symptoms. Cortical atrophy has been reported to be predominantly present in frontoparietal regions (especially in areas along the pre- and postcentral gyrus) and the basal ganglia [2], [4], [5]. However, case studies have also reported atrophy in the temporal and occipital lobes [6], [7], [8]. Regarding clinical symptoms, unilaterally beginning limb apraxia, rigidity, bradykinesia, myoclonus, dystonia and alien limb phenomena have consistently been stated [5], [8], [9], [10], [11], [12], while depression, memory and speech-related impairment have been found less frequently [2], [6], [7], [11]. Notably, previous studies have summarized groups of CBS patients irrespective of the initially impaired body side [2], [4], [9], or have not even reported the initially affected body side [10], [11], [13], [14].

Structural and functional studies investigating hemispheric lateralization [15], [16], however, suggest that the two hemispheres are differentially susceptible to age- or disease-related changes [17], [18], [19]. The most frequently reported hemispherical functional difference is the dissociation of verbal and spatial processing - functions, which are lateralized [20], [21]. This functional lateralization tends to be less pronounced in older age, as elderly reveal more bilateral patterns of activation [17], [22]. In addition, Dolcos et al. (2002) reviewed evidence from behavioral and imaging studies for the so-called “right hemi-aging model”, according to which the right hemisphere is assumed to be more sensitive to age-related structural as well as functional changes [23]. Consistent with this model, studies found older subjects to be less impaired in verbal compared to spatial functions [24]. Furthermore, a better recovery of motor functions was reported for women with left-hemispheric stroke compared to women with affection of the non-dominant right hemisphere [18]. These findings point to differences in functional loss between the two hemispheres, and suggest the hypothesis that the side of clinical onset might be related to the neuropathological and behavioral heterogeneity of the clinical phenomena and differences in the course of disease.

Therefore, we investigated CBS pathology separately for patients with left- and right-beginning impairment (l-CBS resp. r-CBS), and investigated differences in local brain volume reductions and neuropsychological performance in a cross sectional matched group analysis. Furthermore, we analyzed differences in atrophy and neuropsychological impairment in a longitudinal study of two female patients with l-CBS or r-CBS in order to characterize disease-related changes in individual patients in addition to the cross-sectional design of the first part of the study.

## Methods

### 1.1 Sample

Eight patients diagnosed with CBS according to research criteria [25] (**Appendix S1**) by clinical neurologists of the Neurological Clinic of the Düsseldorf University Hospital, Germany, were assigned to two equally sized groups of right (r-) and left (l-) beginning CBS based on the side of the body that had been reported to be impaired first. For the cross sectional study, they were matched for sex, age (l-CBS: 67.8±9.2 years, r-CBS: 68.2±4.2 years; F = .331, p>.05) and duration of disease (l-CBS: 3.5±1.3 years, r-CBS: 3.8±1.5 years; t = −.253, p>.05). Our original sample included seven more subjects, which had to be excluded due to deviation from matching criteria. Furthermore, a healthy control group (N = 8) comparable to the patient groups in sex and age (64.9±7.6 years) was investigated.

For longitudinal single case investigation, data of patients 1 and 5 was explored at t_0_ and six months later at t_1_. Both females were different in impaired body side (l-CBS resp. r-CBS); they were similar in disease duration (3.8 resp. 4 years) and time interval between initial and follow-up examination (six months). Both patients were included in group analyses as well. The longitudinal investigation (t_0_-t_1_) took place eight months later than the examination used for the cross-sectional group analysis. All patients provided written informed consent to participate. The study was approved by the local ethics committee (“Ethikkommission der Medizinischen Fakultät der Heinrich-Heine-Universität Düsseldorf”) and in accordance with the Declaration of Helsinki.

### 1.2 MRI

MRI was acquired on a 3 Tesla scanner (Siemens Magnetom Trio Tim System, Siemens Medical Solutions; Erlangen Germany) with a standard CP head coil. The pulse sequence was as follows: Sagittal 3D T1 magnetization-prepared rapid acquisition gradient echo (MPRAGE) sequence, repetition time (TR)  = 2.3 s, echo time (TE)  = 2.98 ms, slice thickness  = 1 mm, number of slices  = 192, flip angle  = 9°, field of view (FOV)  = 256 mm and 256×256 matrix.

### 1.3 Neuropsychological assessment

All patients underwent an extensive neuropsychological assessment of attention, memory, speech, executive functions, visuo-perceptual abilities as well as motor functions including apraxia. In addition, patients' handedness was investigated and a dementia and depression screening was carried out (**Appendix S2**). Neuropsychological test data was taken from the same point in time as the MRI; when deviating from MRI, the earliest neuropsychological examination after MRI was reported.

### 1.4 Deformation field morphometry

Inter-individual differences in local brain volume were examined by means of deformation field morphometry (DFM), a method that enables quantification of local differences in brain volume between groups of subjects (for details see [26]). Region-based DFM measures of cytoarchitectonically defined brain regions of the Jülich-Düsseldorf cytoarchitectonic atlas [27] were calculated, supplemented by macroanatomically defined structures of the MNI Template [28] in brain regions, where cytoarchitectonical areas have not yet been mapped (**Appendix S3**). Each subject's MR data was registered to the T1-weighted single subject brain of the MNI template (“Colin27 brain”). After non-linear registration, deformation fields of each brain were calculated and transformed to “local volume ratio” (LVR) maps [26]. LVR maps indicate voxel-wise structural differences between the individual and template brain. Volumes of cyto- and macroanatomical regions of interest were calculated by summing LVR-values of the LVR-map corresponding to that region. To describe individual disease progression for single cases, follow-up images were registered to the patient's initial image, and voxel-wise volume differences between t_0_ and t_1_ were computed for the same regions of interest.

### 1.5 Statistical analysis of volume differences

Differences in local brain volume between patients with l-CBS, r-CBS and controls were explored using principal component analysis (PCA), subsequent MANOVA and discriminant analysis. First, the various areal volumes were assigned to 12 macroanatomical groups of topographically related brain regions (topography-groups (TGs)) (**Appendix S4**) and analyzed via PCA. As a result, areal volumes within each TG were structured into a set of relevant principal components (PCs), which were set as to account for more than 90 percent of the variance in brain volume within the TG. The selected PCs were then tested for group differences by means of MANOVA, including multivariate statistics and pairwise post hoc comparisons of patient groups and controls. Finally, discriminant analysis revealed the accuracy of differentiating between patient groups and controls on the basis of the first two PCs within each TG.

To evaluate differences in the amount of local volume deviation between l-CBS and r-CBS patients, volumetric differences were explored as follows: Cyto- and macroanatomical areal volume measures within significant macroanatomical TGs were bootstrapped (n = 2000) and averaged for each patient group and controls. Averaged areal volumes of the control group were subtracted from averaged volumes of the corresponding areas of each patient group. The difference in volume measures was then divided by the SD of the control group in order to indicate the amount of deviation of patients' local brain volume. Brain regions were considered to indicate disease-related atrophy when the average local volume of a patient group was outside the 99 percent confidence interval of the control group.

### 1.6 Longitudinal changes in brains of patients 1 and 5 over six months

To assess individual disease progression, regional atrophy at t_0_ and changes in local brain volume within six months (from t_0_ to t_1_) were examined in patients 1 and 5. To assess already existing differences in regional atrophy between both cases at the initial examination of the longitudinal study (t_0_), local volume differences of at least four standard deviations compared to the mean of the control group were considered as relevant. In addition to initially present volumetric differences between cases (t_0_), further individual volumetric changes from t_0_ to t_1_ were analyzed. According to Fjell et al (2009), annual volume reductions in healthy aging ranged from 0.24 to 0.84 percent, depending on brain region. Based on these findings, a criterion of two percent was applied as indicative of pathological atrophy [29].

## Results

### 2.1. Differences in atrophy patterns between CBS-patients and controls

**Figure 1:**
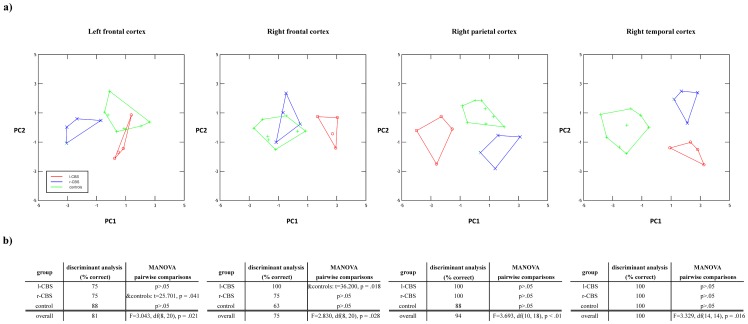
Statistical analysis on brain volumetric differences between l-CBS, r-CBS and healthy controls. a) Two-dimensional canonical analysis of components of significant topographical groups. Euclidean distances were used as measure for differences in local brain volume between CBS groups and controls. b) Results of discriminant analysis and MANOVA.

Significant differences were found in local brain volume between l-CBS, r-CBS and controls bilaterally in the frontal cortex, as well as in parietal and temporal regions of the right hemisphere based on results of multivariate statistics on selected principal components of each macroanatomical topography group (**Appendix S4**). Discriminant analysis revealed that, on the basis of the first two components within each TG, at least 75 percent of the subjects could be classified correctly (**Figure 1**). With respect to left-hemispheric areal volume, r-CBS patients were best discriminable from l-CBS patients and controls on the basis of PC1 (volume in premotor cortex as most contributing region). In addition, 75 percent of l-CBS patients were differentiable from the control group based on PC2 (volume in orbitofrontal cortex most contributing). Within the right hemisphere, l-CBS patients were perfectly discriminable from r-CBS patients and controls on the basis of regional volume in the superior frontal gyrus. Moreover, l-CBS patients were discriminable from r-CBS patients by volume in the intraparietal sulcus. Both, l-CBS and r-CBS patients were differentiable from the control group by volume in the secondary somatosensory and entorhinal cortex.

### 2.2. Differences in atrophy patterns between l-CBS and r-CBS patients

**Figure 2:**
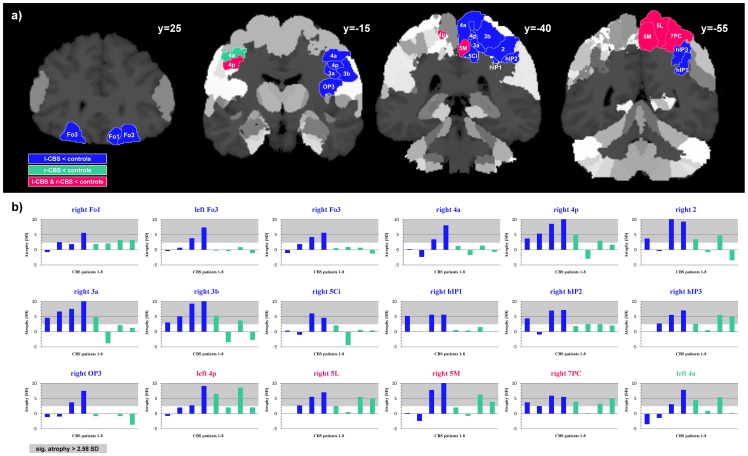
Atrophy patterns in l-CBS and r-CBS groups compared to controls. a) Volumetric differences between patients and controls overlaid on coronal sections of the JuBrain cytoarchitectonic atlas. Colored areas illustrate significant volume reductions in l-CBS (blue), r-CBS (green) or in both CBS groups (pink). b) Each patient's volume reduction (in SD) compared to the control group. Patients 1 to 8 are listed from left to right according to Table 1, with l-CBS patients in blue and r-CBS patients in green. For brain areas that were found to be atrophic in group statistics, titles of the diagrams are printed in blue for the l-CBS group, in green for the r-CBS group and pink for overlap between the l-CBS and r-CBS group. Significant volume reductions of 2.58 SD or more are shaded in grey.

Further exploration of the amount of local volume differences between l-CBS and r-CBS groups revealed correspondences and differences in the pattern of atrophy (**Figure 2a**). Primary motor areas were affected in both groups in the respective hemisphere contralateral to the impaired limb. However, atrophy in the l-CBS group affected more brain regions and extended partly bilaterally to primary motor and orbitofrontal regions. Moreover, primary and secondary somatosensory areas of the contralateral hemisphere were affected in l-CBS only, in addition to the intraparietal sulcus. In contrast to l-CBS, superior parietal areas of the ipsilateral hemisphere showed more atrophy in the r-CBS group (**Appendix S5**). Each patient's contribution to brain morphological group differences is visualized in **Figure 2b**.

### 2.3. Differences in neuropsychological performance between l-CBS and r-CBS patients

Most r-CBS patients revealed a balanced neuropsychological performance throughout the cognitive domains, while most l-CBS patients revealed lower than average performance on object perception, word fluency, verbal and figural memory. Furthermore, l-CBS patients varied highly in their apraxia score (one patient could not make use of his affected limb at all, another hardly revealed any impairment compared to the healthy limb), but were, over all, as impaired as r-CBS patients, whose performance did not vary as much (**Table 1**).

**Table 1:** Neuropsychological test performance of l-CBS and r-CBS patients.

Function	l-CBS				r-CBS			
	Raw score (t-score)				Raw score (t-score)			
	Patient 1	Patient 2	Patient 3	Patient 4	Patient 5	Patient 6	Patient 7	Patient 8
*Examination*								
MRI	02/2011	09/2009	04/2010	03/2008	05/2009	07/2012	01/2009	05/2011
Neuropsychology	02/2011	03/2010	04/2010	03/2010	03/2010	07/2012	04/2010	05/2011
*Memory*								
Verbal working memory	10 (48)	10 (48)	9 (45)	8 (38)	10 (48)	11 (55)	6 (25)	9 (45)
Visual-spatial working memory (max. 18)	-	7	5	unable	9	10	10	9
Verbal episodic memory	32 (41.9)	41 (40.8)	22 (29.7)	12 (29.7)	63 (63.4)	48 (58.1)	21 (29.7)	48 (44.6)
Figural memory	7 (40)	9 (50)	4 (30)	unable	11 (60)	8 (45)	unable	12 (67)
*Attention*								
Processing speed	35.6 (43)	36 (43)	94 (25)	unable	79 (25)	30 (45)	154.5 (25)	66.25 (36)
*Executive functioning*								
Affinity of interference	-	27 (43)	32 (45)	unable	18.4 (50)	-	unable	27.7 (50)
*Language*								
Word fluency (min. 30)	-	27	18	9	45	43	unable	20
Naming (max. 120)	117	117	116	unable	120	120	unable	120
*Perception*								
Object (max. 20)	14	18	10	unable	20	19	unable	19
Space (max. 10)	9	7	6	unable	10	10	unable	9
*Dementia*								
MMST (max. 30)	-	28	21	13	29	30	21	30
MDRS (max. 144)	130	129	126	144	141	-	unable	144
*Apraxia*								
FAST (max. 67)	left: 53	left: 0	left: 24	left: unable	left: 51	left: 50	left: unable	left: 54
	right: 55	right: 57	right: 42	right: unable	right: 32	right: 32	right: unable	right: 32

-  =  missing; minimum (min.) resp. maximum (max.) performance is shown as reference where standard values are not available. Values deviating from normative data are underlined.

### 2.4 Longitudinal changes in brain atrophy of patients 1 and 5

**Figure 3:**
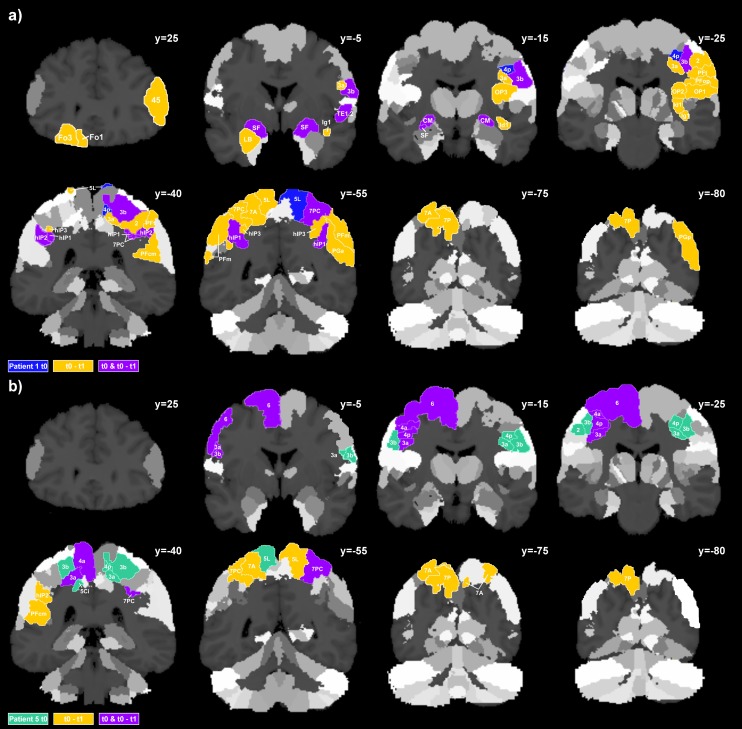
Atrophy patterns in l-CBS and r-CBS single cases. Areal volume reductions in CBS patients at t_0_ and from t_0_ to t_1_ overlaid on coronal sections of the JuBrain cytoarchitectonic atlas: Colored areas illustrate a) atrophy of four or more SD in patient 1 and b) patient 5.

The two cases differed in the pattern of neural atrophy, both at the initial point of examination and during disease progression. In patient 1 (l-CBS), atrophy was mainly characterized by bilaterally affected primary somatosensory areas, superior parietal, intraparietal and temporal regions, followed by extending atrophy in orbitofrontal areas in the course of the disease (**Figure 3a**). In contrast, patient 5 (r-CBS) revealed bilateral atrophy predominantly in the premotor, primary motor and somatosensory cortex, followed by atrophy extending bilaterally to the superior parietal cortex and a worsening of already affected motor areas (**Figure 3b**). All atrophic brain regions, including the amount of deviation from the control group (at t_0_) and the amount of atrophy during disease progression (t_0_-t_1_) are listed in **Appendix S6**.

Neuropsychological profiles differed for the single cases, both at the initial examination and during further disease progression. The cognitive status in patient 1 was mainly characterized by perceptual and memory deficits in the beginning, which worsened in the course of the disease, whereas her apraxia worsened only slightly. In contrast, patient 5 revealed a relatively stable, average neuropsychological performance during both examinations, whereas her apraxia had already been pronounced at t_0_ and further worsened in the following six months (**Table 2**).

**Table 2:** Changes in neuropsychological performance in the course of six months for patient 1 and patient 5.

Function	Patient 1 (t_0_ –t_1_)		Patient 5 (t_0_ – t_1_)	
	Raw score (t-score)		Raw score (t-score)	
*Memory*				
Verbal working memory	12 (60)	11 (55)	10 (48)	11 (55)
Visual-spatial working memory (max. 18)	8	-	9	10
Verbal episodic memory	27 (39.5)	39 (46.8)	57 (70.3)	54 (65.4)
Figural memory	8 (40)	10 (55)	-	10 (55)
*Attention*				
Processing speed	53.0 (33)	-	79.0 (25)	83.0 (25)
*Executive functioning*				
Affinity of interference	34.9 (38)	25.2 (45)	18.4 (50)	21.1 (48)
*Language*				
Word fluency (min. 30)	29	31	45	36
Naming (max. 120)	117	119	120	120
*Perception*				
Object (max. 20)	9	7	20	20
Space (max. 10)	9	10	10	10
*Dementia*				
MMST (max. 30)	23	22	29	27
MDRS (max. 144)	135	130	141	131
*Apraxia*				
FAST (max. 67)	left: 54	left: 46	left: 51	left: 50
	right: 57	right: 52	right: 32	right: 3

Table legend is equivalent to that of Table 1.

## Discussion

In this prospective study, patients with l-CBS and r-CBS differed in their pattern of brain atrophy. Furthermore, longitudinal case studies suggest differences in progression of neuronal degeneration and cognitive decline for l-CBS and r-CBS patients. Taken together, results support the notion about differences in hemispheric susceptibility to disease-related changes.

### Methodological considerations

Overall, the interpretation and generalizability of the present study's cross-sectional and longitudinal results is limited based on the small sample size. On the one hand, this is caused by our single center approach, where all patients were investigated at the same location, by the same MRI scanner and the same examiner. The advantage of this approach is a reduction of investigation errors. On the other hand, the sample size was reduced by our matching procedure: In order to decrease inter-subject variability in this rare clinical syndrome, patients were matched for sex, age and disease duration in an optimal manner. This resulted in a small, but homogeneous sample, in which differences in brain morphometry and neuropsychological performance could be investigated without embedded corrections in group statistical methods.

Furthermore, we were not able to guarantee that there were not different subtypes of CBS included in our groups [30], as most of our patients are still alive and no post-mortem diagnoses were available. But each of the patients in this study met the research criteria for probable or possible CBS [25], and the contribution to brain morphological group results of every single patient (**Figure 2b**) appeared to be homogeneous for the subgroups of l-CBS and r-CBS patients.

In addition, there was a time difference between the MRI and the neuropsychological investigation of six to 24 months in two l-CBS and two r-CBS enclosed in this study. This was caused by the constitution of the patient during the day of investigation. Therefore, interpretation of these data was done in a very conservative way. In spite of these considerations, we are sure that data of this study give important contribution to a better understanding of the diversity of CBS by separating l-CBS from r-CBS patients.

### Differences in atrophy patterns and neuropsychological performance between l-CBS and r-CBS

Both, l-CBS and r-CBS groups revealed atrophy in the primary motor cortex contralateral to the affected body side. Volume reductions in these brain regions had previously been described in studies that did not separate l-CBS and r-CBS patients [2], [4], [5] and are supposed to be responsible for deficits in voluntary movement [1]. The l-CBS, but not the r-CBS group, revealed further atrophy in the primary and secondary somatosensory cortex, as well as in superior and intraparietal areas contralateral to the affected body side. Parts of primary somatosensory cortex are involved in processing and discrimination of shape (area 2) [31], [32], which was investigated during neuropsychological testing by the visuo-perceptual subtest for object perception and can be described as impaired in l-CBS. Affection of other primary somatosensory areas (areas 3a and 3b) is associated with deficits in somatosensory responsiveness and discrimination of moving stimuli [33]; volume reductions in superior parietal cortex (area 5) are linked to hand and arm movement, the manipulation of objects as well as somatosensory and visuomotor integration [34], [35]. The involvement of primary somatosensory and superior parietal areas together most likely reflects cortical sensory deficits [1] that were found to be present especially in l-CBS patients. Further, the additional involvement of the intraparietal sulcus is likely to worsen visuo-perception and cortical sensory deficits and impair proprioception [1] in l-CBS patients, as it has been shown to be involved in perceptual-motor coordination, e.g. in reaching and grasping (hIP1), as well as in visual attention and the manipulation of hand movement (hIP2) [36].

Similar to the differences found between l-CBS and r-CBS groups, single case patient 1 (l-CBS) and patient 5 (r-CBS) revealed a different pattern of cognitive impairment and neuronal atrophy, at the first and second point in time during examination. In accordance with the l-CBS group, patient 1 initially showed atrophy in primary motor and somatosensory areas, superior parietal cortex and bilaterally in the intraparietal sulcus. Likewise, neuropsychological performance was in accordance with group results and revealed especially visuo-perceptual and cortical sensory deficits, besides apraxia. Beyond that, however, extended bilateral atrophy was found in the amygdala, basal ganglia and thalamic regions, in which areal volume reductions were strongest. After six months, atrophy further extended to orbitofrontal and bilaterally to already affected areas in the superior and inferior parietal cortex. This atrophy pattern was consistent with impaired performance on neuropsychological tests of memory [37], [38] on the one hand and a slight worsening of motor functions on the other [39]. In contrast to patient 1, atrophy in patient 5 was most pronounced in left primary motor areas and the premotor cortex, although bilateral primary somatosensory and superior parietal areas were slightly affected at the initial point of examination as well. Accordingly, motor-related impairment (e.g. deficits in hand and arm movement, somatosensory responsiveness and proprioception) was especially predominant in patient 5 initially and still worsened remarkably with disease progression. Symptoms were accompanied by further atrophy in primary motor and somatosensory areas as well as left-hemispheric superior parietal volume reductions in the course of six months.

Overall, besides overlapping patterns of atrophy in the primary motor and primary somatosensory cortex for l-CBS and r-CBS, atrophy in l-CBS affected relatively more brain areas and further extended to the orbitofrontal cortex in group and single case analyses as well. Furthermore, r-CBS patients appeared to be primarily affected in motor-related functioning, whereas beyond that, l-CBS patients seemed to be additionally impaired in perception and memory. Although single cases revealed additional areal volume reductions compared to CBS groups, the extent of initial and progressed atrophy pattern as well as distinctive symptoms of patients 1 and 5 (apraxia, perceptual and memory deficits versus primarily impairment of motor functioning) still appeared characteristic for the respective CBS group. These findings emphasize the importance of investigating groups of patients with l-CBS or r-CBS separately. The fact that CBS groups and single cases were well matched for age and disease duration allows the assumption that differences in cognitive performance and brain atrophy patterns are attributable to the side of clinical onset. The differences might be interpreted as an earlier manifestation of neurodegeneration with faster progression in cases where the left body side and contralateral hemisphere had initially been affected and suggest differences in hemispheric susceptibility to disease-related changes. Even if the sample size due to the incidence of CBS is small, and especially more longitudinal data of neural degeneration and cognitive impairment are desirable, this study documents the importance of considering the initially affected body side in CBS in clinical routine, as this differentiation may help to clarify the clinical picture, diagnosis and individual prognosis of CBS patients.

## Supporting Information

Appendix S1 **Clinical description of included CBS patients.** + (present); according to Armstrong et al. (2013), six patients were diagnosed with probable and 2 patients with possible corticobasal syndrome (the latter marked with an asterisk *). The presence of all symptoms was clinically acquired in each patient's case history at the moment of first visit. The presence of the alien limb phenomenon was confirmed if the patient reported feelings of strangeness, and of spontaneous, uncontrollable and involuntary limb movement that might also include co-activation of the other limb.
(DOC)

Appendix S2 **Description of neuropsychological tests listed according to different cognitive domains.**
(DOC)

Appendix S3 **Cytoarchitectonic areas according to the respective publications.**
(DOC)

Appendix S4 **Principal components and factor loadings within statistically significant macroanatomical topography groups.** PC (principal component), TG (topography group), ICBM (macroanatomically defined structures of the MNI Template). Factor loadings contributing most to the individual component are printed in bold.
(DOC)

Appendix S5 **Amount of deviation (in SD) in local brain volume of CBS groups compared to controls.**
(DOC)

Appendix S6 **Amount of local brain volume deviation (in SD) from the control group at t_0_ and amount of atrophy (in %) from t_0_ to t_1_ in CBS single cases.**
(DOC)

Data S1 (XLSX)
