# Supplementary material for: Neuropsychological and Brain Volume Differences in Patients with Left- and Right-Beginning Corticobasal Syndrome
Source: PLoS One. 2014 Oct 30;9(10):e110326. doi: 10.1371/journal.pone.0110326 (PMC4214821; doi:10.1371/journal.pone.0110326)
Supplement: Appendix S1 — Clinical description of included CBS patients. + (present); according to Armstrong et al. (2013), six patients were diagnosed with probable and 2 patients with possible corticobasal syndrome (the latter marked with an asterisk *). The presence of all symptoms was clinically acquired in each patient's case history at the moment of first visit. The presence of the alien limb phenomenon was confirmed if the patient reported feelings of strangeness, and of spontaneous, uncontrollable and involuntary limb movement that might also include co-activation of the other limb. (DOC) [file pone.0110326.s001.doc]

Appendix S1.

Clinical description of included CBS patients.

|  | l-CBS | | | | r-CBS | | | |
| --- | --- | --- | --- | --- | --- | --- | --- | --- |
|  | Patient 1 | Patient 2 | Patient 3* | Patient 4 | Patient 5 | Patient 6 | Patient 7* | Patient 8 |
| Gender | w | m | w | m | w | m | m | w |
| Age (in years) | 57 | 64 | 74 | 77 | 63 | 67 | 68 | 72 |
| Disease duration (in years) | 1.5 | 4 | 2 | 2 | 2 | 1 | 5 | 1.5 |
| Family history | - | - | - | - | - | - | - | - |
| Asymmetry | + | + | + | + | + | + | + | + |
| Rigidity/ akinesia | + | + | + | + | + | + | + | + |
| Dystonia | + | + | - | + | + | - | + | + |
| Myoclonus | - | - | - | - | + | + | - | + |
| Apraxia | + | + | + | + | + | + | + | + |
| Cortical sensory deficit | + | + | + | - | - | + | - | + |
| Alien limb syndrome | - | - | + | + | + | - | - | + |

+ (present); according to Armstrong et al. (2013), six patients were diagnosed with probable and 2 patients with possible corticobasal syndrome (the latter marked with an asterisk *). The presence of all symptoms was clinically acquired in each patient’s case history at the moment of first visit. The presence of the alien limb phenomenon was confirmed if the patient reported feelings of strangeness, and of spontaneous, uncontrollable and involuntary limb movement that might also include co-activation of the other limb.
