# Supplementary material for: Neuropsychological and Brain Volume Differences in Patients with Left- and Right-Beginning Corticobasal Syndrome
Source: PLoS One. 2014 Oct 30;9(10):e110326. doi: 10.1371/journal.pone.0110326 (PMC4214821; doi:10.1371/journal.pone.0110326)
Supplement: Appendix S2 — Description of neuropsychological tests listed according to different cognitive domains. (DOC) [file pone.0110326.s002.doc]

Appendix S1.

Description of neuropsychological tests listed according to different cognitive domains.

| Function | Test | Reference | Description |
| --- | --- | --- | --- |
| *Memory* |  |  |  |
| Verbal working memory | Digit-span test (DST) Subtest of the Nürnberger-Alters Inventar |  | Sum-score indicating performance in the correct (forward and backward) recall of a numerical series (Digit- Span (score sum)) |
| Visual-spatial working memory | Corsi Block Tapping Test (CBT) |  | Sum-score indicating performance in correctly (forward and backward) tapped blocks (previously demonstrated by the instructor) (CBT (score sum)) |
| Verbal episodic memory | Verbaler Gedächtnistest (VGT) |  | Sum of free recalled items within 5 trials of a list containing 15 words (orally presented to the subject) (VGT(learning)) |
| Figural memory | Figure test (FT) Subtest of the Nürnberger-Alters Inventar |  | Number of recognized (previously shown) figures (of ten figures, each shown between 3 distractor figures) |
| *Attention* |  |  |  |
| Processing speed | Zahlen-Verbindungs-Test (ZVT-T1) German subtest of the Nürnberger-Alters Inventar | Similar to Trail Making Test Version A | Time (in seconds) needed to connect randomly arranged digits in ascending order via drawing lines |
| *Executive functioning* |  |  |  |
| Affinity of interference | Farb-Wort-Interferenz Test (FWIT) | Similar to  and | Time lag (in seconds) between performance in naming the ink-color of colored bars and naming the ink-color that color-words were printed in (FWIT 3-2) |
| *Language* |  |  |  |
| Word fluency | Controlled oral word association test (COWAT) |  | Total number of produced words beginning with the letter F within one minute |
| Naming | Naming –Subtests of the Aachener Aphasie Test |  | Number of incorrect named drawings of objects or scenes |
| *Perception* |  |  |  |
| Object | Incomplete letters (IL) |  | Number of correct named letters (which were masked with a pattern of randomly assigned dots) |
| Space | Number location (NL) Subtests of the Visual Object and Space Perception Battery |  | Number of correctly identified location of numbers (which corresponded to certain position of a dot on a reference card) |
| *Motor skills* |  |  |  |
| Apraxia | Florida Apraxia Screening Test (FAST) |  | Number of correctly imitated gestures with the right and left hand |
| *Dementia* |  |  |  |
|  | Mini-mental-status-test (MMST) German version |  | Screening test for cognitive impairment |
|  | Mattis Dementia Rating Scale (MDRS) German version |  | Screening test for cognitive impairment with focus on motor impairment |
| *Depression* |  |  |  |
|  | Beck Depression Inventory (BDI-II) German version |  | Self-report inventory indicating a possible depression |

1. Oswald WD, Fleischmann UM (1997) Nürnberger-Alters-Inventar (NAI). Testinventar & NAI-Testmanual und Textband (4. Auflage). Göttingen: Hogrefe.

2. Schelling D (1997) Block-Tapping-Test. Frankfurt: Swets Test Service GmbH.

3. Lux S, Hartje W, Reich C, Nagel C (2012) VGT: Verbaler Gedächtnistest: Bielefelder Kategorielle Wortlisten. Göttingen: Hogrefe

4. Morris JC, Heyman A, Mohs RC, Hughes JP, van Belle G, et al. (1989) The Consortium to Establish a Registry for Alzheimer's Disease (CERAD). Part I. Clinical and neuropsychological assessment of Alzheimer's disease. Neurology 39: 1159-1165.

5. Bäumler G (1985) Farbe-Wort-Interferenztest (FWIT). Göttingen: Hogrefe.

6. Stroop JR (1935) Studies of interference in serial verbal reactions. Journal of Experimental Psychology 18: 643-662.

7. Huber W, Poeck K, Weniger D, Willmes K (1983) AAT: Aachener Aphasie Test. Göttingen: Hogrefe.

8. Warrington EK, James M (1991) The Visual Object and Space Perception Battery. Bury St. Edmunds, England: Thames Valley Test Company.

9. Rothi LJG, Heilman KM (1984) Acquisition and retention of gestures by apraxic patients. Brain and Cognition 3: 426-437.

10. Folstein MF, Folstein SE, Mchugh PR (1975) Mini-Mental State - Practical Method for Grading Cognitive State of Patients for Clinician. Journal of Psychiatric Research 12: 189-198.

11. Mattis S (1988) Dementia Rating Scale professional manual. Odessa, FL: Psychological Assessment Resources.

12. Hautzinger M (1991) The German Version of the Beck Depression Inventory in Clinical Use. Der Nervenarzt 62: 689-696.
