# Supplementary material for: Neuropsychological and Brain Volume Differences in Patients with Left- and Right-Beginning Corticobasal Syndrome
Source: PLoS One. 2014 Oct 30;9(10):e110326. doi: 10.1371/journal.pone.0110326 (PMC4214821; doi:10.1371/journal.pone.0110326)
Supplement: Appendix S3 — Cytoarchitectonic areas according to the respective publications. (DOC) [file pone.0110326.s003.doc]

**Appendix S1.**

**Cytoarchitectonic areas according to the respective publications.**

| Frontal cortex | Orbitofrontal cortex (area Fo1, Fo2, Fo3) |
| --- | --- |
|  | Broca’s region (area 44 and 45) |
|  | Premotor cortex (area 6) |
|  | Motor cortex (area 4a and 4p) |
| Parietal cortex | Primary somatosensory cortex (3a, 3b, 1, area 2) |
|  | Secondary somatosensory cortex (area OP1-4) |
|  | Superior parietal cortex (area 5L, 5M, 5Ci, 7A, 7PC, 7M, 7P) |
|  | Inferior parietal cortex (area PFop, PFt, PF, PFm, PFcm, PGa, PGp) |
|  | Intraparietal sulcus (area hIP1, hIP2, hIP3) |
| Temporal cortex | Superior temporal cortex (area TE 1.0, TE 1.1, TE 1.2, TE 3) |
|  | Hippocampus (area CA *cornu ammonis*, FD *fascia dentate*, HATA *hippocampal-amygdaloid transition area*) |
|  | Entorhinal cortex |
|  | Amygdala (area CM *centromedial*, LB *laterobasal*, SF *superficial*) |
| Insula | Posterior insula (area Ig1, Ig2 *granular insula*, Id1 *dysgranular insula*) |

1. Henssen AG (2013) Zytoarchitektonische Analyse des orbitofrontalen Kortex beim Menschen.

2. Amunts K, Schleicher A, Bürgel U, Mohlberg H, Uylings HBM, et al. (1999) Broca's region revisited: Cytoarchitecture and intersubject variability. The Journal of Comparative Neurology 412: 319-341.

3. Geyer S (2004) The microstructural border between the motor and the cognitive domain in the human cerebral cortex. Advances in anatomy, embryology, and cell biology 174: I-VIII, 1-89.

4. Geyer S, Ledberg A, Schleicher A, Kinomura S, Schormann T, et al. (1996) Two different areas within the primary motor cortex of man. Nature 382: 805-807.

5. Geyer S, Schleicher A, Zilles K (1999) Areas 3a, 3b, and 1 of Human Primary Somatosensory Cortex: 1. Microstructural Organization and Interindividual Variability. NeuroImage 10: 63-83.

6. Grefkes C, Geyer S, Schormann T, Roland P, Zilles K (2001) Human Somatosensory Area 2: Observer-Independent Cytoarchitectonic Mapping, Interindividual Variability, and Population Map. NeuroImage 14: 617-631.

7. Eickhoff SB, Schleicher A, Zilles K, Amunts K (2006) The Human Parietal Operculum. I. Cytoarchitectonic Mapping of Subdivisions. Cerebral Cortex 16: 254-267.

8. Eickhoff SB, Amunts K, Mohlberg H, Zilles K (2006) The Human Parietal Operculum. II. Stereotaxic Maps and Correlation with Functional Imaging Results. Cerebral Cortex 16: 268-279.

9. Scheperjans F, Eickhoff SB, Hömke L, Mohlberg H, Hermann K, et al. (2008) Probabilistic Maps, Morphometry, and Variability of Cytoarchitectonic Areas in the Human Superior Parietal Cortex. Cerebral Cortex 18: 2141-2157.

10. Scheperjans F, Hermann K, Eickhoff SB, Amunts K, Schleicher A, et al. (2008) Observer-Independent Cytoarchitectonic Mapping of the Human Superior Parietal Cortex. Cerebral Cortex 18: 846-867.

11. Caspers S, Geyer S, Schleicher A, Mohlberg H, Amunts K, et al. (2006) The human inferior parietal cortex: Cytoarchitectonic parcellation and interindividual variability. NeuroImage 33: 430-448.

12. Caspers S, Eickhoff SB, Geyer S, Scheperjans F, Mohlberg H, et al. (2008) The human inferior parietal lobule in stereotaxic space. Brain Structure and Function 212: 481-495.

13. Choi H, Zilles K, Mohlberg H, Schleicher A, Fink GR, et al. (2006) Cytoarchitectonic identification and probabilistic mapping of two distinct areas within the anterior ventral bank of the human intraparietal sulcus. The Journal of Comparative Neurology 495: 53-69.

14. Morosan P, Rademacher J, Schleicher A, Amunts K, Schormann T, et al. (2001) Human Primary Auditory Cortex: Cytoarchitectonic Subdivisions and Mapping into a Spatial Reference System. NeuroImage 13: 684-701.

15. Morosan P, Schleicher A, Amunts K, Zilles K (2005) Multimodal architectonic mapping of human superior temporal gyrus. Anatomy and Embryology 210: 401-406.

16. Amunts K, Kedo O, Kindler M, Pieperhoff P, Mohlberg H, et al. (2005) Cytoarchitectonic mapping of the human amygdala, hippocampal region and entorhinal cortex: intersubject variability and probability maps. Anatomy and Embryology 210: 343-352.

17. Amunts K, Fink G (2005) Introduction - The convergence of brain structure and function - (Presented at the) Second Vogt Brodmann Symposium in Julich, Germany. Anatomy and Embryology 210: 335-335.
