# Supplementary material for: Neuropsychological and Brain Volume Differences in Patients with Left- and Right-Beginning Corticobasal Syndrome
Source: PLoS One. 2014 Oct 30;9(10):e110326. doi: 10.1371/journal.pone.0110326 (PMC4214821; doi:10.1371/journal.pone.0110326)
Supplement: Appendix S4 — Principal components and factor loadings within statistically significant macroanatomical topography groups. PC (principal component), TG (topography group), ICBM (macroanatomically defined structures of the MNI Template). Factor loadings contributing most to the individual component are printed in bold. (DOC) [file pone.0110326.s004.doc]

Appendix S4.

Principal components and factor loadings within statistically significant macroanatomical topography groups

| *Frontal cortex (TG1) left*  *(F=3.043, df (8, 20), p = .021)* | PC1 | | | | PC2 | | | | PC3 | | | | PC4 | | |
| --- | --- | --- | --- | --- | --- | --- | --- | --- | --- | --- | --- | --- | --- | --- | --- |
| Broca’s area (area 44) | 0.345 | | | | -0.458 | | | | 0.151 | | | | 0.162 | | |
| Broca’s area (area 45) | 0.239 | | | | -0.552 | | | | -0.290 | | | | 0.487 | | |
| Orbitofrontal cortex (area Fo1, Fo2) | 0.261 | | | | **0.553** | | | | -0.123 | | | | 0.192 | | |
| Orbitofrontal cortex (area Fo3) | 0.304 | | | | 0.317 | | | | **-0.565** | | | | 0.314 | | |
| Primary motor cortex (area 4) | 0.427 | | | | -0.026 | | | | 0.277 | | | | -0.317 | | |
| Premotor cortex (area 6) | **0.447** | | | | 0.024 | | | | 0.311 | | | | 0.004 | | |
| Middle frontal gyrus ^ICBM^ | 0.350 | | | | -0.136 | | | | -0.508 | | | | **-0.692** | | |
| Superior frontal gyrus ^ICBM^ | 0.397 | | | | 0.243 | | | | 0.357 | | | | 0.146 | | |
| *Cumulative proportion* | *0.542* | | | | *0.767* | | | | *0.872* | | | | *0.920* | | |
| *Frontal Cortex (TG1) right*  *(F=2.830, df (8, 20), p = .028)* | PC1 | | | | PC2 | | | | PC3 | | | | PC4 | | |
| Broca’s area (area 44) | -0.369 | | | | -0.310 | | | | -0.113 | | | | 0.453 | | |
| Broca’s area (area 45) | -0.271 | | | | **-0.593** | | | | -0.121 | | | | -0.375 | | |
| Orbitofrontal cortex (area Fo1, Fo2) | -0.278 | | | | 0.323 | | | | **-0.787** | | | | -0.023 | | |
| Orbitofrontal cortex (area Fo3) | -0.288 | | | | 0.564 | | | | -0.013 | | | | 0.006 | | |
| Primary motor cortex (area 4) | 0.331 | | | | 0.326 | | | | 0.548 | | | | -0.246 | | |
| Premotor cortex (area 6) | -0.407 | | | | -0.021 | | | | -0.040 | | | | -0.474 | | |
| Middle frontal gyrus ^ICBM^ | -0.401 | | | | -0.028 | | | | 0.785 | | | | **0.604** | | |
| Superior frontal gyrus ^ICBM^ | **-0.442** | | | | -0.148 | | | | 0.131 | | | | -0.064 | | |
| *Cumulative proportion* | *0.589* | | | | *0.767* | | | | *0.853* | | | | *0.904* | | |
| *Parietal Cortex (TG2) right*  *(F=3.693, df (10, 18), p < .01)* | PC1 | | | PC2 | | | PC3 | | | | PC4 | | | PC5 | |
| Intraparietal sulcus (area hIP1) | **0.352** | | | 0.063 | | | -0.107 | | | | -0.226 | | | -0.326 | |
| Intraparietal sulcus (area hIP2) | **0.352** | | | 0.040 | | | -0.082 | | | | 0.123 | | | -0.208 | |
| Intraparietal sulcus (area hIP3) | 0.287 | | | 0169 | | | -0.180 | | | | **-0.452** | | | -0.273 | |
| Inferior parietal lobule (area PF) | 0.289 | | | -0.066 | | | -0.314 | | | | 0.329 | | | -0.144 | |
| Inferior parietal lobule (area PG) | 0.280 | | | 0.357 | | | -0.092 | | | | 0.180 | | | 0.050 | |
| Primary somatosensory cortex (area 1) | 0.236 | | | -0.265 | | | 0.289 | | | | 0.353 | | | -0.363 | |
| Primary somatosensory cortex (area 2) | 0.340 | | | -0.198 | | | 0.199 | | | | 0.144 | | | -0.123 | |
| Primary somatosensory cortex (area 3) | 0.303 | | | -0.012 | | | 0.294 | | | | 0.015 | | | 0.402 | |
| Secondary somatosensory cortex (area OP1) | -0.041 | | | **-0.440** | | | -0.418 | | | | 0.020 | | | 0.033 | |
| Secondary somatosensory cortex (area OP2) | 0.202 | | | -0.430 | | | -0.045 | | | | -0.396 | | | 0.163 | |
| Secondary somatosensory cortex (area OP3) | 0.215 | | | -0.410 | | | -0.238 | | | | -0.001 | | | 0.390 | |
| Secondary somatosensory cortex (area OP4) | -0.041 | | | 0.214 | | | **-0.600** | | | | 0.329 | | | 0.126 | |
| Superior parietal lobule (area 5) | 0.297 | | | 0.180 | | | 0.176 | | | | 0.237 | | | **0.449** | |
| Superior parietal lobule (area 7) | 0.258 | | | 0.317 | | | -0.098 | | | | -0.350 | | | 0.215 | |
| *Cumulative proportion* | *0.454* | | | *0.635* | | | *0.763* | | | | *0.853* | | | *0.900* | |
| *Temporal Cortex (TG3) right*  *(F=3.329, df (14, 14), p = .016)* | | PC1 | PC2 | | | PC3 | | PC4 | | PC5 | | PC6 | | | PC7 |
| Amygdala (CM) | | -0.302 | 0.340 | | | 0.135 | | -0.009 | | -0.161 | | **0.507** | | | 0.109 |
| Amygdala (LB) | | -0.305 | 0.301 | | | -0.026 | | -0.305 | | -0.328 | | -0.337 | | | 0.063 |
| Amygdala (SF) | | -0.417 | 0.131 | | | 0.339 | | -0.003 | | -0.154 | | 0.113 | | | 0.027 |
| Auditory cortex (area TE1.0, TE1.1, TE1.2) | | 0.061 | -0.083 | | | **0.583** | | -0.402 | | 0.242 | | 0.288 | | | -0.003 |
| Auditory cortex (area TE3) | | 0.364 | 0.048 | | | 0.324 | | -0.168 | | 0.105 | | -0.134 | | | 0.577 |
| Entorhinal cortex | | **-0.450** | 0.038 | | | -0.003 | | 0.135 | | -0.038 | | -0.301 | | | 0.334 |
| Hippocampus (CA, FD, HATA) | | -0.047 | 0.363 | | | -0.356 | | -0.442 | | 0.157 | | 0.307 | | | -0.118 |
| Hippocampus (Subiculum) | | -0.241 | 0.028 | | | -0.278 | | -0.288 | | **0.590** | | -0.239 | | | 0.187 |
| Parahippocampal gyrus ^ICBM^ | | -0.335 | -0.227 | | | 0.079 | | 0.122 | | 0.522 | | 0.091 | | | -0.123 |
| Inferior temporal gyrus ^ICBM^ | | -0.034 | -0.386 | | | -0.297 | | -0.172 | | -0.203 | | 0.287 | | | **0.610** |
| Middle temporal gyrus ^ICBM^ | | -0.085 | **-0.443** | | | -0.248 | | -0.225 | | -0.200 | | 0.245 | | | -0.162 |
| Superior temporal gyrus ^ICBM^ | | -0.082 | -0.318 | | | 0.191 | | **-0.534** | | -0.213 | | -0.348 | | | -0.275 |
| Fusiform gyrus ^ICBM^ | | -0.340 | -0.368 | | | 0.157 | | 0.203 | | -0.001 | | 0.051 | | | 0.040 |
| *Cumulative proportion* | | *0.294* | *0.505* | | | *0.629* | | *0.752* | | *0.832* | | *0.889* | | | *0.931* |

PC (principal component), TG (topography group), ICBM (macroanatomically defined structures of the MNI Template). Factor loadings contributing most to the individual component are printed in bold.
