# Supplementary material for: Neuropsychological and Brain Volume Differences in Patients with Left- and Right-Beginning Corticobasal Syndrome
Source: PLoS One. 2014 Oct 30;9(10):e110326. doi: 10.1371/journal.pone.0110326 (PMC4214821; doi:10.1371/journal.pone.0110326)
Supplement: Appendix S5 — Amount of deviation (in SD) in local brain volume of CBS groups compared to controls. (DOC) [file pone.0110326.s005.doc]

Appendix S5.

Amount of deviation (in SD) in local brain volume of CBS groups compared to controls.

| Hemisphere | Region | Area | l-CBS | r-CBS |  |
| --- | --- | --- | --- | --- | --- |
| contralateral | frontal | Fo1 | 2.6 | < 2.58 |  |
|  |  | Fo3 | 3.02 | < 2.58 |  |
|  |  | 4a | 2.83 | 2.77 |  |
|  |  | 4p | 7.56 | 4.65 |  |
|  | parietal | 2 | 2.7 | < 2.58 |  |
|  |  | 3a | 8.36 | < 2.58 |  |
|  |  | 3b | 7.67 | < 2.58 |  |
|  |  | 5Ci | 2.79 | < 2.58 |  |
|  |  | 5L | 4.22 | < 2.58 |  |
|  |  | 5M | 4.66 | < 2.58 |  |
|  |  | 7PC | 4.65 | < 2.58 |  |
|  |  | hIP1 | 4.32 | < 2.58 |  |
|  |  | hIP2 | 4.75 | < 2.58 |  |
|  |  | hIP3 | 3.01 | < 2.58 |  |
|  |  | OP3 | 2.72 | < 2.58 |  |
|  | temporal | fusiform gyrus^ICBM^ | 3.34 | < 2.58 |  |
| ipsilateral | frontal | Fo3 | 3.29 | < 2.58 |  |
|  |  | 4p | 3.78 | < 2.58 |  |
|  | parietal | 5L | < 2.58 | 3.26 |  |
|  |  | 5M | < 2.58 | 2.76 |  |
|  |  | 7PC | < 2.58 | 3.02 |  |
