# Supplementary material for: Neuropsychological and Brain Volume Differences in Patients with Left- and Right-Beginning Corticobasal Syndrome
Source: PLoS One. 2014 Oct 30;9(10):e110326. doi: 10.1371/journal.pone.0110326 (PMC4214821; doi:10.1371/journal.pone.0110326)
Supplement: Appendix S6 — Amount of local brain volume deviation (in SD) from the control group at t0 and amount of atrophy (in %) from t0 to t1 in CBS single cases. (DOC) [file pone.0110326.s006.doc]

Appendix S6.

Amount of local brain volume deviation (in SD) from the control group at t_0_ and amount of atrophy (in %) from t_0_ to t_1_ in CBS single cases.

| Hemisphere | Region | Area | Cases t_0_ (in SD) | | Cases t_1_ (in %) | |
| --- | --- | --- | --- | --- | --- | --- |
|  |  |  | Patient 1 | Patient 5 | Patient 1 | Patient 5 |
| contralateral | frontal | 4a | < 4 | 5.10 | < 2 | 2 |
|  |  | 4p | 5.58 | 7.17 | < 2 | 2 |
|  |  | 6 | < 4 | 4.50 | < 2 | 2 |
|  |  | 45 | < 4 | < 4 | 2 | < 2 |
|  |  | Inferior frontal gyrus^ICBM^ | < 4 | < 4 | 2 | < 2 |
|  |  | Middle frontal gyrus^ICBM^ | < 4 | < 4 | 2 | < 2 |
|  | parietal | 2 | < 4 | 4.90 | 3 | < 2 |
|  |  | 3a | < 4 | 6.89 | 2 | 2 |
|  |  | 3b | 4.27 | 5.56 | 2 | < 2 |
|  |  | 5Ci | < 4 | 4.20 | 2 | < 2 |
|  |  | 5L | < 4 | 4.21 | < 2 | < 2 |
|  |  | 5M | 4.50 | < 4 | < 2 | < 2 |
|  |  | 7PC | 4.08 | < 4 | 2 | 2 |
|  |  | 7A | < 4 | < 4 | < 2 | 2 |
|  |  | 7P | < 4 | < 4 | < 2 | 2 |
|  |  | PF | < 4 | < 4 | 2 | < 2 |
|  |  | PFcm | < 4 | < 4 | 3 | < 2 |
|  |  | PFop | < 4 | < 4 | 3 | < 2 |
|  |  | PFt | < 4 | < 4 | 3 | < 2 |
|  |  | PGa | < 4 | < 4 | 2 | < 2 |
|  |  | PGp | < 4 | < 4 | 3 | < 2 |
|  |  | hIP1 | 4.05 | < 4 | 5 | < 2 |
|  |  | hIP2 | 4.74 | < 4 | 5 | 2 |
|  |  | hIP3 | < 4 | < 4 | 3 | < 2 |
|  |  | OP1 | < 4 | < 4 | 2 | < 2 |
|  |  | OP2 | < 4 | < 4 | 2 | < 2 |
|  |  | OP3 | < 4 | < 4 | 2 | < 2 |
|  | temporal | Amygdala (CM) | 9.13 | < 4 | < 2 | < 2 |
|  |  | Amygdala (SF) | 8.56 | < 4 | < 2 | < 2 |
|  |  | parahippocampal gyrus^ICBM^ | 6.03 | 4.29 | < 2 | < 2 |
|  |  | TE1.2 | 4.85 | < 4 | 2 | < 2 |
|  | insula | Id1 | < 4 | < 4 | 2 | < 2 |
|  |  | Ig1 | < 4 | < 4 | 3 | < 2 |
|  | basal ganglia | putamen^ICBM^ | 13.83 | < 4 | < 2 | < 2 |
|  |  | nucleus accumbens^ICBM^ | 11.43 | < 4 | < 2 | < 2 |
|  |  | ventral globus pallidus | 13.78 | < 4 | < 2 | < 2 |
|  |  | caudate nucleus^ICBM^ | 9.35 | < 4 | 2 | < 2 |
|  | thalamus | anterior group of nuclei | 13.33 | < 4 | < 2 | 2 |
|  |  | medial group of nuclei | 13.76 | < 4 | < 2 | < 2 |
|  |  | ventral group of nuclei | 12.95 | < 4 | < 2 | < 2 |
|  | substantia nigra | pars compacta | 10.05 | < 4 | 2 | < 2 |
|  |  | pars reticularis | 9.74 | < 4 | < 2 | < 2 |
|  | periaqueductal grey |  | < 4 | < 4 | < 2 | 4 |
| ipsilateral | frontal | Fo1 | < 4 | < 4 | 3 | < 2 |
|  |  | Fo3 | < 4 | < 4 | 2 | < 2 |
|  |  | 4p | < 4 | 5.35 | < 2 | < 2 |
|  | parietal | 3a | < 4 | 5.62 | < 2 | < 2 |
|  |  | 3b | < 4 | 5.65 | < 2 | < 2 |
|  |  | 5Ci | < 4 | < 4 | 2 | < 2 |
|  |  | 5L | < 4 | < 4 | 2 | 2 |
|  |  | 7PC | < 4 | 4.46 | 3 | 2 |
|  |  | 7A | < 4 | < 4 | 3 | 2 |
|  |  | 7P | < 4 | < 4 | 2 | < 2 |
|  |  | PFm | < 4 | < 4 | 2 | < 2 |
|  |  | hIP1 | 5.59 | < 4 | 5 | < 2 |
|  |  | hIP2 | 4.23 | < 4 | 5 | < 2 |
|  |  | hIP3 | < 4 | < 4 | 3 | < 2 |
|  | temporal | Amygdala (CM) | 8.69 | < 4 | 3 | < 2 |
|  |  | Amygdala (LB) | < 4 | < 4 | 3 | 2 |
|  |  | Amygdala (SF) | 6.70 | < 4 | 2 | < 2 |
|  |  | parahippocampal gyrus^ICBM^ | 5.04 | < 4 | < 2 | < 2 |
|  | basal ganglia | putamen^ICBM^ | 13.57 | < 4 | < 2 | < 2 |
|  |  | nucleus accumbens^ICBM^ | 9.83 | < 4 | < 2 | < 2 |
|  |  | ventral globus pallidus | 8.98 | < 4 | < 2 | < 2 |
|  |  | caudate nucleus^ICBM^ | 5.40 | < 4 | < 2 | < 2 |
|  | thalamus | anterior group of nuclei | 8.12 | < 4 | < 2 | 2 |
|  |  | medial group of nuclei | 11.28 | < 4 | < 2 | < 2 |
|  |  | ventral group of nuclei | 10.04 | < 4 | < 2 | < 2 |
|  | substantia nigra | pars compacta | 8.71 | < 4 | < 2 | < 2 |
|  |  | pars reticularis | 7.63 | < 4 | < 2 | < 2 |
|  | nucleus ruber^ICBM^ |  | 6.54 | < 4 | < 2 | < 2 |
|  | periaqueductal grey |  | < 4 | < 4 | < 2 | 2 |
